# Supplementary material for: Analysis of PPARγ Signaling Activity in Psoriasis
Source: Int J Mol Sci. 2021 Aug 10;22(16):8603. doi: 10.3390/ijms22168603 (PMC8395241; doi:10.3390/ijms22168603)
Supplement: Supplementary file 1 [file ijms-22-08603-s001.zip › Supplemental materials_Analysis of PPARg signaling activity in psoriasis/Pathway models/Models images and html files/PPARG expressed in cells assotiated with psoriasis/116005.html]

dermis


# Tissue dermis

|  |  |
| --- | --- |
| URN | urn:agi-ncimtissue:C0011646 |
| Total Entities | 5 |
| Connectivity | 2457 |
| Name | dermis |

---

|  |  |
| --- | --- |
| ChildConcepts | acellular dermal matrix |
|  | dermoepidermal junction |
|  | papillary dermis |
|  | reticular dermis |
|  | glomus body |

---

|  |  |
| --- | --- |
| Pathway | PPARG expressed in cells assotiated with psoriasis |

---

|  |  |
| --- | --- |
| MedScan ID | 6000420 |

---

|  |  |
| --- | --- |
| Alias | corium |
|  | dermises |
|  | dermis |

---

|  |  |
| --- | --- |
| NCIm ID | C0011646 |

---
